# Knowledge, attitudes, and practices of Lebanese licensed dietitians regarding hyperphosphatemia management in patients undergoing hemodialysis in a Lebanese Governorate

**DOI:** 10.1186/s12882-024-03936-w

**Published:** 2025-02-14

**Authors:** H. Mannan, E. Issa, R. Attieh, Y. Sacre

**Affiliations:** 1 Translational Health Research Institute and School of Medicine, Western Sydney University, Campbelltown campus, NSW, Australia; 2 Department of Nutrition and Dietetics, Holy Spirit University of Kaslik, Jounieh, Lebanon

**Keywords:** Behavioral change, Dietary counseling, Dietitians, HD, Phosphorus knowledge

## Abstract

**Background:**

Hyperphosphatemia is deemed to be an asymptomatic silent killer, its high prevalence in patients undergoing hemodialysis (HD) is correlated mainly with malnutrition and mortality. Dietitian’s renal nutrition education has a major impact on chronic kidney disease patients’ knowledge, and attitude towards nutrition guidelines. However, a large number of factors are acting as barriers to the appropriate practices of Lebanese dietitians in renal therapy. This study evaluated the knowledge, attitudes, and practices (KAP) of licensed dietitians (LDs) regarding hyperphosphatemia management in patients undergoing HD.

**Methods:**

A total of 408 LDs from Mount-Lebanon Governorate in Lebanon participated in this study. A 52-item online questionnaire was used to assess nutritional phosphorus’ KAP of all LDs, in compliance with dietetic practices with KDOQI guidelines updated version 2020 and identified the factors preventing dietitians from dealing with renal patients undergoing HD, e.g., nutrition care. The data was analyzed using SPSS version 25.

**Results:**

There was a significant association between KAP levels, and almost all sociodemographic characteristics evaluated. Only 2% of dietitians applied all KDOQI guidelines, 64% attained poor and moderate knowledge, and 60% had a positive attitude towards renal care. Working in a clinical field was a common predictor of positive knowledge (adjusted OR = 2.453, 95% CI 1.244–4.836), positive attitude (adjusted OR = 1.900, 95% CI 1.300–2.541) and positive practice (adjusted OR = 0.192, 95% CI 0.184–0.491) while HD/hospital-based field increased the odds for positive knowledge (adjusted OR = 4.520, 95% CI 1.189–17.182). LDs, compared to registered dietitians, had lower odds of positive knowledge (adjusted OR = 0.390, 95% CI 0.231–0.658) and positive attitude (adjusted OR = 0.270, 95% CI 0.154–0.471). Lack of training was the main reason preventing the appropriate dietetic practices regarding hyperphosphatemia management in patients undergoing HD.

**Conclusion:**

The Ministry of Public Health (MOPH) should be asked to endorse the integration of renal nutrition programs in the Lebanese curriculum, to aid in the empowerment of dietitians from different backgrounds towards renal therapy, in order to enhance the knowledge and attitude regarding nutritional guidelines of poorly supported Lebanese patients undergoing HDhemodialysis. Other stakeholders may include the Syndicate of Dietitians in Lebanon.

**Supplementary Information:**

The online version contains supplementary material available at 10.1186/s12882-024-03936-w.

## What do we already know about this topic?

In Lebanon, HD patients are provided care in both hospitals and private settings with most getting care in hospitals. Studies in Lebanon have explored dietetic practices in HD units and compliance with KDOQI guidelines in a sample of dietitians providing care to patients receiving HD treatment in hospitals only, but not for CKD in general [1]; renal nutrition knowledge in a small sample of licensed dietitians LDs [2]; and the effect of nutrition education on management of osteodystrophy among HD patients [3]. However, there are lack of studies relevant to dietitians providing care outside the hospital setting, e.g., in private practice. The reasons are unknown that prevent clinical registered dietitians (not only hospital-based ones) from dealing with renal patients undergoing HD, because colloquium holders are entitled to do so. Finally, there are not enough studies in other countries too on the topic of study.

## How does your research contribute to the field?

This study is the first to direct knowledge, attitudes, and practices of LDs licensed dietitians regarding hyperphosphatemia-suffering HD patients, and the leading to addressing the compliance with dietetic practices and KDOQI nutrition updated guidelines version 2020. Our study overcomes the limitation of a previous study [1] which explored dietetic practices in HD units in Lebanon but not CKD in general, and that of another small study [2] which examined only renal nutrition knowledge among Lebanese licensed dietitians (LDs) but not attitudes and practice while our study examined all the three aspects—knowledge, attitude and practice using a large sample.

## What are your research’s implications towards theory, practice, or policy?

It filled gaps in nutrition practices by LDs identifying the factors that prevent them from dealing with hyperphosphatemia-suffering patients undergoing HD and thus overcoming the limitation of a previous study on the topic which was not for CKD in general and another study which that didn’t examine practice and attitude but only renal nutrition knowledge among Lebanese LDs In terms of policy, it emphasizes the necessity of building effective training programs, under the supervision of the ministry of public health and the universities in Lebanon, to enhance their long-term behavioral change in collaboration with the Lebanese order of dietitians.

## Introduction

Phosphorus in food products exists in either organic or inorganic form. Organic phosphorus, or naturally occurring phosphorus, can be found in animal and plant foods (such as seeds, nuts, and legumes), having an different intestinal absorption rates of 40–80% and 20–40% respectively [4]. Alternatively, inorganic phosphorus, such as phosphates added to foods during processing, has an absorption rate of greater than 90% [5]. Having excessive phosphate in serum known as hyperphosphatemia, is commonly defined as a serum phosphate ⩾4.5 mg/dl. This is deemed to be an asymptomatic silent killer [6]. Besides anemia and hyperkalemia, hyperphosphatemia is the most frequent metabolic complication in kidney failure (KF). Its prevalence in hemodialysis (HD) patients is as high as 50%, and it is correlated majorly with vascular calcification, mineral dysmetabolism, and malnutrition [7]. The evidence is inconclusive on whether increasing knowledge of the renal diet leads to increased adherence to dietary restrictions and better patient outcomes [8–11]. Evidence suggests that nutrition literacy could predict dietary adherence if components of self-efficacy and self-management are taken into consideration [11].

It is recognized that during the period between 2012 and 2021, the HD scene in Lebanon spotted a notable spurt in the total number of recently added HD units, from 64 to more than 75, related to the annually rapidly increasing number: 4,270 patients who are receiving treatment with chronic HD [12]. Previous studies have consistently shown that renal phosphorus nutritional awareness of patients undergoing HD is very weak when compared to sodium, potassium, and protein knowledge [13]. However, specified strategies are indispensable, to maximize the inclusion of nutrition experts and dietetic coverage in HD units in Lebanon, and to educate, empower, guide, and turn patients' fear into nutritional confidence [3, 14]. In consequence, achieving that requires a solid education and capacity to communicate information as well as competencies in theory-based nutrition counseling (rather than a transfer of information), among other factors, especially when it comes to hyperphosphatemia management [15–17].

Dietitians as experts in renal nutrition care are uniquely qualified for this role. They get to know the renal patients’ food habits and preferences and can work with them to create a meal plan that should be followed when at home or out and about. Sticking to the food, fluid and nutrition plan developed by the renal dietitian is key to managing kidney disease and kidney failure.

It is very important to focus on licensed dietitians (LDs) for a better understanding of knowledge, attitudes, and practices (KAP) among health care professionals [3, 14]. On the other hand, the KAP of LDs regarding hyperphosphatemia-suffering patients undergoing HD remains understudied and unclear worldwide [18]. Although no data investigated their KAP in Lebanon specifically regarding hyperphosphatemia-suffering patients undergoing HD, dietitians’ renal nutrition education has a major impact on chronic kidney disease (CKD) patients’ knowledge and adherence to phosphorus, attitude towards renal nutrition guidelines, and wellbeing [18]. Although the Kidney Disease Outcomes Quality Initiative (KDOQI), founded in the United States, is recognized as the most reliable and professional source adapted for renal therapy, in Lebanon there is uncertainty in its use by LDs. Studies in United States [19] and Lebanon [1] have explored dietetic practices in HD units and compliance with KDOQI guidelines in a sample of dietitians providing care to patients receiving HD treatment in dialysis centres and hospitals respectively, but neither for hyperphosphatemia nor CKD in general. Other studies conducted in hospital settings of in Lebanon have examined renal nutrition knowledge in a small sample of LDs [2], and the effect of nutrition education on the management of osteodystrophy among HD patients [3]. However, there are lack of studies relevant to dietitians providing care outside the hospital setting, e.g., in private practice. It may be noted that in Lebanon, the LDs are those who are graduates of bachelor’s degree in human nutrition and dietetics and are colloquium holders of Lebanese dietetic licensure. They become registered dietitians after they complete at least six months of hospital-dietetic practice and internship and then pass the national and annual nutrition examination. Only the registered dietitians are entitled to treat renal patients undergoing HD. However, the reasons are unknown that prevent them from dealing with renal patients undergoing HD.

But unfortunately, a large number of factors are barricading the appropriate nutritional practices, integration, and involvement of Lebanese dietitians with HD staff [20, 21]. Thus, the following problems arise:

What are the reasons that keep dietitians from dealing with hyperphosphatemia-suffering patients undergoing HD ?

The general aim of this study is thus to evaluate the knowledge, attitudes, and practices (KAP) of LDs regarding hyperphosphatemia management in patients undergoing HD in both hospitals and private settings. The patients they are responsible for treating may not all undergo HD following CKD, but also have other chronic conditions. Also, this study addresses the compliance with dietetic practices and KDOQI nutrition updated guidelines version 2020, and identifies barriers preventing them from dealing with patients undergoing HD. This initiative would consequently empower dietitians from different backgrounds in renal therapy to get integrated into the HD unit’s health care team, enhance the nutritional aspects of poorly supported Lebanese patients undergoing HD and most importantly raise the awareness of their families about the importance of nutrition and lifestyle [15, 22].

In Lebanon, HD units are often hospital-based, but most hospitals have only one dietitian performing all-nutrition related activities in the hospital. The rationale for the current study on the importance of exploring the KAP of LDs not only working in hospitals but also in private practice, lies in the evidence from the research by Karavetian et al. [1] which supported the need to explore LDs providing care outside the hospital setting, e.g., private practice. There is also a low compliance with KDOQI guidelines among dietitians managing HD patients in Lebanese hospitals including an extremely low hospital dietitian-to-patient contact time and the many barriers precluding hospital dietitians from providing nutrition care to patients undergoing HD [1]. These suggest that the patients might also be receiving care in other (private) settings and that there is a fragmented nutrition care delivery system in Lebanon.

## Methods

### Study design

To investigate the research questions, a cross-sectional study was conducted in different districts of Mount-Lebanon governorate in Lebanon, between the end of July and the beginning of August 2021. A large number of factors coupled with appropriate study design are acting as barriers to the appropriate nutritional practices, integration, and involvement of Lebanese dietitians with HD staff, in preventing and treating clinically pertinent aspects, mainly hyperphosphatemia. So, what are the reasons that keep dietitians from dealing with hyperphosphatemia-suffering patients undergoing HD?

### Recruitment procedure

For data collection, the snowball sampling technique was adopted to select licensed and registered dietitians. As it is a listing method, the respondents were also called to help the researcher identify other potential participants, e.g., colleague dietitians in the Mount-Lebanon districts (Jbeil, Keserwan, Metn, Aley, Baabda, and Chouf). An online questionnaire and a consent form, were uploaded on Google Forms. Initially, the link was sent to colleagues and anonymous dietitians, via LinkedIn, Email, WhatsApp, Instagram, and Facebook, and in return, participants were also called to identify other potential dietitians: they were also asked to share the link of the questionnaire with their colleagues. The target sample was 408. The study was performed between the end of July and the beginning of August 2021. Mount Lebanon was chosen among the eight governorates of Lebanon because of the diversity of its residents. According to the Central Administration of Statistics, Mount Lebanon has a population of 22,673 [23]. The eligibility for involvement was being licensed or registered dietitian nutritionists who were colloquium holders of Lebanese dietetic licensure. Colloquium holders are graduates with bachelor’s degrees in human nutrition and dietetics who can apply for the national and annual nutrition examination, after achieving at least six months of hospital-dietetic practice and internship. This examination is approved by the Ministry of Public Health (MOPH). On the other hand, a registered dietitian is an LD who succeeded in the examination of the Commission on Dietetic Registration of the Academy of Nutrition and Dietetics in the United States. So, all LDs are not registered dietitians.

Inclusion criteria were being licensed or registered dietitians having Lebanese citizenship including those working in clinical, hospital/HD and other settings including food service management, community nutrition, research, administration and sales; those working and non-working males and females; and those having different social classes and having different districts in Mount Lebanon. Thus, different educational levels, working fields, abilitiesy, and willingness were expected ofor the participants.

A questionnaire and a consent form were introduced by the aims of this study. Participants were made aware of the anonymity and confidentiality of every contributor in this study. An online survey with several variables was evolved and conjoined with the LDs who gave their consent to take part in this study.

### Study instrument

Before the onset of this project, the questionnaire was developed from a previous one, adapted to meet Lebanon’s criteria, and approved by the Ethics committee of the Faculty of Arts and Sciences, at the Holy Spirit University of Kaslik. A 52-item self-administered online questionnaire was developed and administrated in the English language. It was framed into five major sections. All questionnaire parts were inspired by prior literature led by different researchers [24–28]. The questionnaire is attached in Appendix 1.

The first part of the questionnaire included all sociodemographic data like age, gender, level of education, professional status, field of work, average monthly income, and years of experience. This section was remodeled by making small changes to meet cultural distinctions in Lebanon. Questions were classified as closed-ended with multiple choices with answers at nominal or interval levels. Modifications were the following: eliminating brand name from the original questionnaire [29], state of work replaced by the district of work, including all places of work instead of hospital-based HD dietitians, ignoring the question regarding race and ethnicity since Lebanon is mostly occupied by the Lebanese, and modulating the level of education based on options available in Lebanon.

Knowledge of dietitians was inspired via a mixture of two adapted and modified questionnaires [25–27] to meet the study objectives. Twenty-three questions were concerned with kidney disease, high phosphorus load, phosphate binders, and dietary phosphate restrictions. Out of these questions, 19 were directly related to phosphate (see Appendix 1). Answers were distributed with three distinct responses: yes, no, don’t know.

The attitude of LDs toward hyperphosphatemia-suffering HD patients was assessed through eight questions. These questions were pertaining to the dietitian whether to agree to take nutritional care; ever consulting a patient while referring him/her to another dietitian or a renal specialist; ever referring the patient to another dietitian but he/she apologizing and asking to refer the patient to another renal specialized dietitian; whether one of the main reasons for referring the patient to another dietitian included: shortage or lack of knowledge or motivation, fear from “renal case”, incapacity of handling HD patient due to lack of trainings and information; shortage in available data and not knowing which resource to adopt (KDOQI or Krause and Mahan [29]) and confusion concerning referral resources; shortage in data adapted to Lebanese community concerning high and low phosphorus food; whether thinking that the dietitians were marginalized in renal therapy, and finally whether one of the main reasons that dietitians were the most marginalized in renal therapy included lack of cooperation with nephrologists and contradiction between dietitians’ and nephrologists’ opinions. Although Krause and Mahan (2021) [29] is the textbook adopted by the majority of nutrition programs in Lebanon for nutrition therapy courses, Mount Lebanon being a low socioeconomic governorate, most (*n* = 228, 55.88%) LDs didn’t have resource to adopt any nutritional tool. This was followed by KDOQI (*n* = 116, 28.4%), Krause and Maher (*n* = 40, 9.8%), preparing the tool with colleagues (*n* = 16, 3.92%) and alone (*n* = 8, 1.96%). For each question, answers were limited to three categorical multiple choices (yes, no, don’t know).

Practices toward nutritional assessment were assessed via seven questions, and awareness concerning the compliance with the updated version 2020 of the KDOQI guideline for nutrition in CKD therapy practices [25]. Examples of the questions on practice were, “In your practice, is 7-Point Subjective global assessment (SGA) considered as the best way for initial assessment of nutritional status? In your practice, is the use of a 3-day food record considered as an alternative method to assess dietary intake?” Answers were categorized between positive and negative practices. For each question, answers are limited to 3 categorical multiple choices (yes, no, don’t know). Additionally, the percentage of guidelines applied by each licensed dietitian (LD) and their compliance with KDOQI guidelines were determined.

The last part of the questionnaire concerned the interest of the LDs in the incorporation of renal specialization in the Lebanese universities’ curriculum and identifying the reasons that prevent dietitians from dealing with HD patients. The last section of the questionnaire on the renal specialization included yes/no type questions on interest in renal specialization; whether wanted to continue degrees in renal specialization in Lebanon, but couldn't due to lack of specialization; and the initiative to register in an online renal course, trainings, and seminars. Also, there was a question on the number of dialysis patients the LDs have been or are now responsible for, the source of patient education material that was used, a yes/no question on whether a bigger focus should be given to renal disease management in the nutrition curriculum, i.e., incorporation of renal specialization in Lebanese universities curriculum, and finally, the reasons that are preventing the LDs to deal with HD patients.

### Statistical analyses

Data were analyzed using SPSS software, version 25. The sample size of this study (*n* = 408) for a population size of *N* = 2743 (Mount-Lebanon, 2014–2021) were all adults aged between 21 and 55 + years based on the Syndicate of Dietitians, the Ministry of Public Health (MOPH) database, was calculated using the method by Thompson (1987) [30]. This assumed a 5% simultaneous type 1 error, and a 5% margin of error (interval of half-width) for estimating each unknown population proportion. Descriptive analysis was performed for demographic variables age, educational level, gender, average monthly income, years of experience, professional status and field of work which were all categorical except for age and years of experience which were continuous. These were represented as frequencies and percentage counts while knowledge, attitude and practice scores were represented as mean ± standard error. For knowledge, answers were scored 1 for each correct answer and 0 for a wrong answer. The total score was expressed out of 23, where 0–12 indicated poor knowledge, 13–16 indicated moderate knowledge, and 17–23 indicated good knowledge [25]. For performing binary logistic regression, good knowledge was recorded as ‘positive knowledge’ while poor and moderate knowledge collapsed as ‘negative knowledge’. While for attitude a mark above or equal to 5 out of 8 is an indicator of positive attitude, a score below 5 out of 8 is an indicator of negative attitude [26]. For practice, the correct answers were coded 1 indicating positive practice when the score was between 4 and 7 out of 7, while wrong answers were coded 0 indicating negative practice when the score was between 0 and 3 out of 7 [25].

For descriptive analysis of continuous variables knowledge, attitude and practice scores, we calculated mean ± SE. Data were outlined as frequencies and percentages for descriptive analysis of categorical variables sex, age groups, education levels, maximum experience of 2 years with CKD, interest in renal specialization, and reasons that might hinder dietitians from dealing with hyperphosphatemia suffering- HD patients.

The Chi-squared test was adopted to test the association between two categorical variables. The Pearson correlation coefficient was used to untangle the association between two quantitative variables. For multivariate analysis, the logistic regression was used to predict a binary dependent variable, positive knowledge (yes vs no), positive attitude (yes vs no) and positive practice (yes vs no). The predictors considered were: professional status, field of work, years of experience, education level, sex and age. They were found significant when *p* ≤ 0.05. Only the predictors found significant are reported in the results tables.

## Results

A total of 408 LDs from the Mount-Lebanon governorate responded and were recruited across different areas in the Mount-Lebanon governorate.

### Sociodemographic and educational characteristics

Out of 408 total participants, 91.2% were females and the majority 86.3% were young and aged between 21 and 34. About 48.0%, 44.1% and 7.8% were bachelor’s, master’s and PhD degree holders, respectively. One-third (*n* = 136, 33.3%) had income between 675,000 and 1,500,000 Lebanese Pounds followed by between 1,500,000 and 3,000,000 Lebanese Pounds (*n* = 108, 26.5%). Results are presented in Table 1.

**Table 1:** Respondent’s sociodemographic characteristics (*N* = 408)

	Number	%
**Gender**		
• Female	372	91.2
• Male	36	8.8
**Age**		
• 21–34	352	86.3
• 35–44	28	6.9
• 45–55	16	3.9
• 55 +	12	2.9
**Level of education**		
• Bachelor’s degree BS	196	48.0
• Master’s degree MS	180	44.1
• PhD degree	32	7.8
**Professional status**		
• Licensed dietitians LD	272	66.7
• Registered dietitian RD	136	33.3
**Field of Work**		
• Clinical dietitian	148	36.3
• Hemodialysis or hospital-based dietitian	28	6.9
• Food service	40	9.8
• Public health or community nutrition	40	9.8
• Freelance or working from home	84	20.6
• Others (Research, administrative, sales)	68	16.7
**Years of experience**		
• 0–2	212	52.0
• 3–5	96	23.5
• 6–10	56	13.7
• 10 +	44	10.8
**Monthly income**		
• Less than 675,000 LBP	56	13.7
• Between 675,000 LBP and 1,500,000 LBP	136	33.3
• Between 1,500,000 LBP and 3,000,000 LBP	108	26.5
• Between 3,000,000 LBP and 5,000,000 LBP	68	16.7
• More than 5,000,000 LBP	40	9.8

### Professional characteristics

These include professional status, the field of work, years of experience, knowledge assessment, attitude assessment, practice assessment, interest in renal specialization, and reasons that might hinder LDs from dealing with hyperphosphatemia-suffering-HD patients. The results are presented in Table 1.

### Professional status

Only 33.33% of the participants were registered dietitians and the remaining 66.67% were only LDs.

### Field of work

Most (*n* = 148, 36.6%) were clinical dietitians followed by freelance or working from home (*n* = 84, 20.6%); research, administrative and sales (*n* = 68, 16.7%); food service (*n* = 40, 9.8%);

public health or community nutrition (*n*=40, 9.8%) and HD or hospital-based dietitian (*n*=28, 6.9%).

### Years of experience

About half (*n*=212, 52%) of the participants had a maximum experience of 2 years with CKD.

### Knowledge assessment

**Fig. 1:**
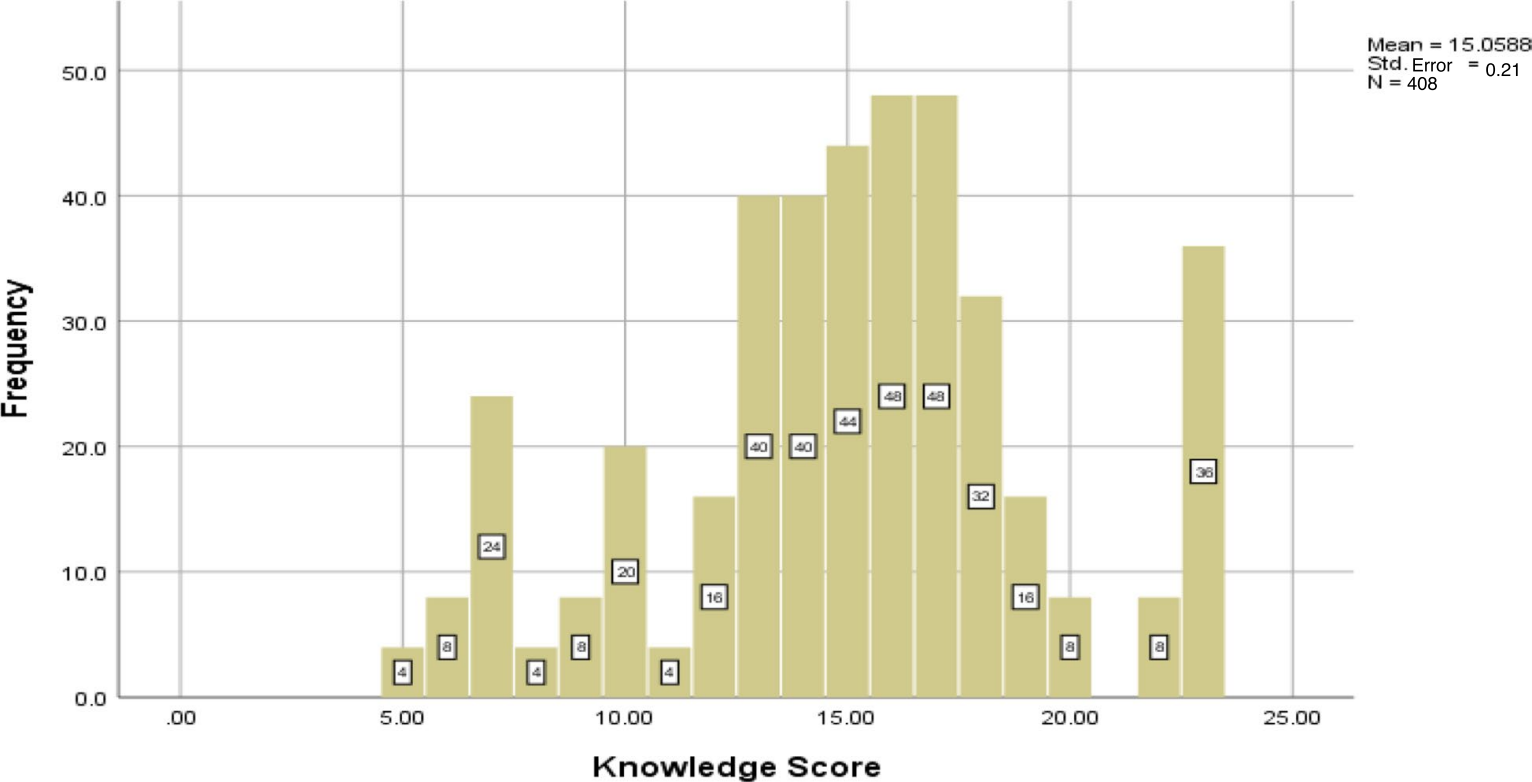
Participant’s knowledge about hyperphosphatemia of chronic kidney disease

The mean knowledge score for participants was 15.05 ± 0.21 (see Fig. 1). About 56.86% of participants had moderate and poor knowledge levels while the remaining. 43.14% had a good knowledge level.

Most of the participants (90.2%) were aware of chronic renal failure, optimal ways to control an adequate blood phosphorus level, and optimal blood phosphorus level. However, 82.4%, 66.7%, 61.8% and 52.9% were unaware of phosphorus pyramid food categories, high phosphorus load consequences, the right time to take phosphate binders, and strategies to reduce dietary phosphorus intake. Of the latter, 52% were responsible for controlling phosphate blood levels in the first place, and 50% of drinks were low in phosphorus.

The association of sociodemographic factors with the knowledge level using the chi-squared test showed that except for gender, there was a significant association between them all and knowledge level (all p-values < 0.001). These are shown in Table S1. Based on binary logistic regression analysis only clinical dietitians and hospital/HD-based dietitians were significant predictors of a positive knowledge outcome (see Table 2 for description of covariates and Table 3 for results). Those working in hospital/HD had the highest 4.52 times higher odds (95% CI 1.189–17.182) of a positive knowledge, followed by those working in the clinical field who had 2.453 times higher odds (95% CI 1.244—4.836), whileen the reference for each group was working in other fields. Being an LD compared to being a registered dietitian was associated with 60.1% lower odds (adjusted OR = 0.390, 95% CI 0.231–0.658) of a positive knowledge outcome.

**Table 2:** Description of predictors used in multivariate logistic regression analysis

Covariates	Variable type	Categories
Gender	Categorical (dummy)	1 = Female, 0 = Male
Age	Continuous	**–-**
**Level of Education**		
Master’s degree level education	Categorical (dummy)	0 = Bachelor’s degree, 1 = Master’s degree
Phd degree level education	Categorical (dummy)	0 = Bachelor’s degree, 1 = PhD degree
**Field of Work**		
Hemodialysis/Hospital	Categorical (dummy)	0 = Others (research, administrative, sales), 1 = Haemodialysis/Hospital
Clinical field	Categorical (dummy)	0 = Others (research, administrative, sales), 1 = Clinical
Public health or community nutrition	Categorical (dummy)	0 = Others (research, administrative, sales), 1 = Public health or community nutrition
Food service	Categorical (dummy)	0 = Others (research, administrative, sales), 1 = Food service
Freelance or working from home	Categorical (dummy)	0 = Others (research, administrative, sales), 1 = Freelance or working from home
**Professional status**	Categorical (dummy)	0 = registered, 1 = licensed
**Years of experience**	Continuous	**–-**
**Monthly income**	Continuous	**–-**

**Table 3:** Significant predictors of participant’s positive knowledge, positive attitude and positive practice about hyperphosphatemia using binary logistic regression

Positive Knowledge				
**Independent Variables**	**Coefficient**	**SE**	**Adjusted Odds Ratio**	**95% CI**
**Field of Work**				
Clinical Field*	0.897	0.346	2.453	1.244–4.836
Hemodialysis/hospital based field*	1.508	0.681	4.520	1.189–17.182
**Professional status**				
Licensed Dietitian	-0.941	0.266	0.390	0.231–0.658
**Positive Attitude**				
**Independent Variables**	**Coefficient**	**SE**	**Adjusted Odds Ratio**	**95% CI**
Gender*	1.243	0.631	3.466	1.006–11.943
**Field of Work**				
Clinical Field*	0.642	0.274	1.900	1.300–2.541
Public health education related field*	-1.697	0.529	0.183	0.065–0.516
Years of experience*	0.588	0.199	1.800	1.219–2.657
Age*	-2.553	0.601	0.078	0.024–0.253
**Professional status**				
Licensed Dietitian	-1.311	0.284	0.270	0.154–0.471
**Positive Practice**				
**Independent Variables**	**Coefficient**	**SE**	**Adjusted Odds Ratio **	**95% CI**
**Field of Work**				
Clinical Field*	-1.650	0.217	0.192	0.184–0.491
**Level of Education**				
Master’s Degree*	1.173	0.251	3.232	1.976–5.285
PhD Degree*	1.543	0.734	4.679	1.110–19.720

Reference categories: ‘Registered dietitian’ for the variable professional status, ‘Male' for the variable gender, ‘Other fields’ for the variable field of work, ‘Bachelor’s Degree’ for the variable level of education

*SE* is standard error of regression coefficient; *CI* is confidence interval

^*﻿^*p*-value ≤ 0.05

### Attitude assessment

The mean attitude score for participants was 4.76 ± 0.10. About 57.84% of dietitians showed a positive attitude toward theend-stage renal disease. One-third of the participants (33.3%) received HD patients and referred them to arenal specialized nutrition care. About 63.7% believed that lack of cooperation with nephrologists, a contradiction between dietitians' and nephrologists' opinions wasere one of the main reasons for referring patients to a specialized dietitian.

More than 70% of LDs agreed that they are marginalized in renal therapy, and the prime reasons for referring patients were: lack of knowledge, lack of motivation, fear ofrom “renal case”, incapacity of handling HD patients due to lack of training and information, shortage in data available and not knowing which resource to adapt (KDOQI or Krause and Mahan [29]), confusion concerning referral resources, and shortage in data adapted to Lebanese community concerning high and low phosphorus food.

The respondent’s knowledge score was not significantly correlated with the attitude score (r= 0.588, *p*-value>0.05). Based on the chi-squared test a significant association was found between age, gender, level of education, professional status, work field, years of experience, monthly income and attitude level (each *p*-value<0.001). These are shown in Table S1. Following binary logistic regression analysis, working in the clinical field, being of the female gender, and increased years of experience were associated with higher odds of a positive attitude (each *p*-value<0.05) (see Table 2 for description of covariates and Table 3 for results). The odds ratios were 1.900 (95% CI 1.300-2.541), 3.466 (95% CI 1.006-11.943) and 1.800 (95% CI 1.219-2.657), respectively. Working in the fields of public health nutrition; being an LD and age were associated with lower odds of positive attitude. The odds ratios were 0.183 (95% CI 0.065-0.516), 0.270 (95% CI 0.154-0.471) and 0.078 (95% CI 0.024-0.253), respectively.

### Practice assessment

The mean practice score for participants was 3.81 ± 0.07. Over half of the participants (54.9%) stated a positive practice. More than half, that is, 68.6%, 69.6%, and 55.9% stated negative practices in nutritional status assessment, body composition assessment, and multivitamins supplementations, respectively. The majority of participants were in compliance with guidelines regarding energy (74.5%) and protein (61.8%), had positive practices regarding alternative methods for dietary intake assessment (53.9%), and factors that should be taken into consideration when assessing dietary intake of HD patients (86.3%). These results are shown in Table 4.

**Table 4:** Licensed dietitians’ practices regarding hyperphosphatemia suffering hemodialysis patients

Variable	Positive practice (%)	Negative practice (%)
1. Assessment of the nutritional status for hemodialysis patients (Guideline 1)	128 (31.4%)	280 (68.6%)
2. Most valid body composition assessment for hemodialysis patients (Guideline 2)	124 (30.4%)	284 (69.6%)
3. Alternative method(s) for dietary intake assessment for hemodialysis patients (Guideline 3)	220 (53.9%)	188 (46.1%)
4. Factors that should be taken into consideration when assessing dietary intake of hemodialysis patients (Guideline 4)	352 (86.3%)	56 (13.7%)
5. Daily protein requirements for hemodialysis patient suffering from diabetes (Guideline 5)	252 (61.8%)	156 (38.2%)
6. Daily energy requirements for hemodialysis patients who are metabolically stable (Guideline 6)	304 (74.5%)	104 (25.5%)
7. Multivitamin supplementation for individuals with adequate vitamin intake (Guideline 7)	176 (43.1%)	228 (55.9%)
**Overall Percentage**	**54.9%**	**45.1%**

Even though a great number of participants (52%) seemed to be educated having Master’s and Doctoral degrees (see Table 1), only 8 (2%) dietitians were in compliance with all KDOQI guidelines (see Table 5).

**Table 5:** Percentage implementation of all the updated KDOQI 2020 guidelines in the dietetic practices

Practice Score = Guidelines Score	Number (n) implementer of each guideline	Percent (%) implementer of each guideline
0.00	8	2.0
1.00	12	2.9
2.00	48	11.8
3.00	116	28.4
4.00	80	19.6
5.00	92	22.5
6.00	44	10.8
7.00	8	2.0

Practice score 0 indicates that dietitians were in conformity with all KDOQI guidelines

The applicant’s knowledge score was linearly associated with the practice score (Pearson correlation, r = 0.657, *p*-value < 0.001); whereas a positive practice was correlated with increased positive knowledge. The mean score of participants’ knowledge; mean ± SE (15.06 ± 0.21) was higher among those with positive practices; mean ± SE (3.81 ± 0.07) concerning hyperphosphatemia in the end-stage renal disease. Based on the chi-squared test a significant association was found between age, level of education, work field, years of experience, monthly income and attitude level (each *p*-value < 0.001)(see Table S1). The logistic regression analysis of factors with positive practice found that being clinical dietitian and having higher education were associated with lower and higher odds of a positive practice (each *p*-value < 0.05). The odds ratios for the clinical dietitian, Master’s degree and PhD degree holders were 0.192 (95% CI 0.184–0.491), 1.173 (95% CI 1.976–5.285) and 1.542 (95% CI 1.110–19.720), respectively. The description of covariates is in Table 2 and the results for logistic regression of a positive practice are shown in Table 3.

### Interest in renal specialization

The results of this section are not shown in a table. Almost 58.82% (*n*=240) were interested in renal specialization, and 41.16% (*n*=168) were unconcerned. 45% (*n*=184) of participants wanted to continue their master's degree in renal specialization in Lebanon but couldn’t due to lack of specialization in Lebanon. Most dietitians (60.8%) didn’t take any online courses in renal therapy or registered in training or seminars. One hundred and sixty-four or 40.2% of LDs were responsible for at least 1 HD patient while the remaining 244 or 59.8% weren’t responsible for any HD patient. Out of those responsible for at least 1 HD patient, the highest 116 or 28.2% was responsible for at least 1-10 HD patients. Only 16 dietitians took care of more than 151 patients (3.6%). Few (28.4%) relied on KDOQI, the official resource for kidney therapy [25], the rest (9.8%) adapted Krause and Mahan [29], or prepared it with colleagues (3.92%), and another 1.96% prepared the educational material alone. Ninety-eight percent(*n*=400) of the LDs urged the need that a bigger focus should be given to renal disease management in the nutrition curriculum.

### Reasons that might hinder dietitians from dealing with hyperphosphatemia suffering- HD patients

A noteworthy high percentage agreed with the following barriers: 324 (79.4%) agreed that lack of knowledge,lack of motivation, fear of “renal case”, unable to handle HD patients due to lack of training and information were the main reasons for referring the patient to another dietitian while 48 (11.8%) showed that it was not a reason, and the remaining 36 (8.8%) selected ‘not sure’ or ‘don’t know’. Three-hundred and four (74.5%) dietitians confirmed that shortage in data availability, not knowing which resource to adapt (KDOQI or Krause and Mahan), and confusion concerning referral resources were among the main 38 reasons for referring the patient to another dietitian. Also, the same number of patients admitted that a shortage in data adapted to the Lebanese community concerning high and low-phosphorus food was one of the main reasons for referring the patient to another dietitian. Two-hundred and sixty (63.7%) a conceded lack of cooperation with nephrologists, that is, the contradiction between dietitians' and nephrologists' opinions was also one of the main reasons while 116 (28.4%) denied it and the remaining 32 (7.8%) had no answer about this concern. Two-hundred and eighty-four (69.6%) of dietitians acknowledged that LDs are marginalized in renal therapy while the rest (n=124, 30.4%) didn’t.

### Practical applications

This study is the first to direct knowledge, attitudes, and practices of LDs. regarding hyperphosphatemia-suffering HD patients, which leads to addressing the compliance with dietetic practices regarding KDOQI nutrition updated guidelines version 2020. It sheds light on a new aspect of filling gaps in nutrition practice by identifying the factors that prevent LDs from dealing with hyperphosphatemia-suffering patients undergoing HD. In addition, it emphasizes the necessity of building effective training programs, under the supervision of the mMinistry of pPublic hHealth and the universities in Lebanon, to enhance their long-term behavioral change in collaboration with the syndicate of dietitians.

## Discussion

This study is the first in Lebanon to direct LDs towards knowledge, attitudes, and practices of hyperphosphatemia-suffering HD patients, and to address the compliance with dietetic practices and KDOQI nutrition updated guidelines version 2020. It incorporated LDs working in both hospitals and outside hospitals who were treating not only CKD patients undergoing HD but also those having other chronic conditions. In view of the existing literature for Lebanon [1–3, 18, 19], there are lack of studies relevant to LDs providing care outside the hospital setting, e.g., in private practice. Also, shortage of studies concerning LDs’ knowledge and practices when it comes to hyperphosphatemia, motivated us towards filling the gap in the present literature [13].

Our study sample having about half of the participants with a maximum experience of two years with CKD is similar to that of another study based on the hospital-based dietitians recruited from a variety of nephrology units inof Lebanon [1]. We have found that working in clinical, hospital-based or HD fields, were common predictors of a positive knowledge outcome, positive attitude, and positive practice. Regarding positive knowledge, this is due to the fact that being attested by the Academy of Nutrition and Dietetics, being a registered dietitian (both clinical dietitians and hospital-based or HD-based dietitians are registered dietitians) passing a more advanced detailed colloquium of the United States with evolved curriculum, might involve more information, helping to enhance clinical dietitians’ and hospital-based or HD-based dietitians’ positive knowledge regarding hyperphosphatemia in HD patients. We also found that LDs had a lower likelihood of positive knowledge and positive attitude but not positive practice. Years of experience increased the likelihood of a positive attitude.

This study has found a significant association between positive knowledge, attitude, and practice, and some of the sociodemographic characteristics evaluated. The implications of the sociodemographic characteristics of our study sample and the correlates of KAP regarding hyperphosphaeteamia of HD patients are discussed below.

The majority of female dietitians (91.2%) participating in this study reflect the interest of females in the Nutrition and Dietetics field worldwide due to their increased attention to health maintenance, food safety, security, and the importance of the involvement of the maternal nutritional role in their children's health [31]. We have found that females have a higher likelihood of positive attitude about hyperphosphatemia than their male counterparts. However, sex is not associated with either positive knowledge or positive practice.

The high percentage of the participants (86.3%) aged between 21 and 34 reflects the young nature of career-making appearance for dietitians in the medical healthcare profession in Lebanon. We have found age as a significant predictor of positive attitude but not for positive knowledge and positive practice. Younger dietitians have a lower likelihood of positive knowledge about hyperphosphatemia than those who are older. Our study may indirectly support the finding of another study which found elevated turnover because of work overcharge, overtiredness, and thus work disappointment [32]. We have found participation of high percentage (44.1%) of LDs having a Master’s degree in our sample; which might be related to the increased unemployment status of Bachelor's degree holders in Lebanon following the economic crisis. On the other hand, if the bachelor's degree holders are working, they are disappointed by the depreciation of business owners toward their values and job skills. Thisis one of the reasons why they prefer to pursue their master's degree instead of performing lowly valued work, or working abroad where dietitians are more respected, and recognized as one of most important professions in the multidisciplinary healthcare team. We have found that compared to Bachelor's degree holders those with Master’s and PhD level education have a higher likelihood of positive practice regarding hyperphosphatemia.

Although the employment impact of COVID is a common factor, the economic situation is improving worldwide, and the Lebanese students and graduates are valued abroad [18]. Furthermore, the increased percentage of highly educated dietitians in our study’s sample (MSc and Ph.D.; 52%) reflects the high level of education generally in the Lebanese community. The percentage of LDs (66.7%) in our study is similar to another study conducted in Lebanon where 61.4% were LDs [19]. The low percentage of registered dietitians in our study is directly linked to the incapability of all the LDs to proceed with the RD tests approved by the Academy of Nutrition and Dietetics in the United States (AND). Also, this is related to the lack of economic resources for dietitians, preventing them from travelling and conducting exams outside the country.

This study attested similarity, with another study when describing the association of practice with educational level [33]. Both studies found positive practice to increase with education.

Only one study in Lebanon scrutinized and compared dietetic practices to KDOQI guidelines [21], but for CKD patients only while we assessed this for LDs treating patients having different chronic conditions including CKD. It showed low compliance with KDOQI guidelines similarly to ours. Another study in the United States evaluating the practices of renal dietitians in nephrology units also found low compliance with KDOQI guidelines [27]. Additionally, these studies showed the urgent need for more effort and inspections, to endeavor compatible, easy, accessible, updated guidelines, and avoid relying on unknown resources. Also, these suggested an urgent need for training on KDOQI standards**.**

Dietitians outlining a positive practice had an elevated knowledge level in this research, but it’s crucial to the highpoint that positive knowledge might not all the time convert to positive practice. This result was confirmed well by another study conducted in Jordan [26].

Lack of integration, time and training, marginalization of renal therapy in universities, fear of renal cases, marginalization in the multidisciplinary renal team, and shortage in the support of ministries were recognized as obstacles to dealing with HD patients. Notable similar results in a study conducted in the United States [34] found a shortage of multi-disciplinary support, work overload, and lack of time and ofvalid resources; those statistics conformed to the present data. Dietitians outlined that the shortage of available ready data and the absence of educational adapted material to the Mediterranean diet in Arabic adds to the overburden of using KDOQI guidelines in nutrition renal therapy, in the middle of the massive charge in the dietetic practice in all work fields. This has also been delineated in the literature [35].

Hospital-based treatment should be only HD-based, therefore it should be incorporated with enough knowledge, time, education, or enablement to adapt KDOQI inpatient care [16, 36–39]. Providing nutrition services is not equilibrated with HD patients; all of this is due to the absence of specialization in renal nutrition therapy in the face of increasing numbers of end-stage patients undergoing HD in the Lebanese community [40].

It is now evident that the phosphorus nutrition knowledge of dietitians had an impact on the compliance with clinical practices regarding KDOQI updated version 2020 [41, 42]. A lack of renal nutrition specialization in Lebanon affects the capacity of dealing with HD patients [42]. The outcome of this study has revealed that only 2% (*n*=8) of LDs were in compliance with all KDOQI guidelines, **e**ven though a great number of them (*n*=212, 52%) were educated having Master’s and Doctoral degrees. About 64% attained poor and moderate hyperphosphatemia knowledge. While more than 82.4% were unaware of the topics related to food-related phosphorus such as phosphorus pyramid, food’s phosphorus content, protein ratio, and phosphorus bioavailability, only 36 LDs were informed regarding all phosphorus’ nutrition aspects. But, on the other hand, most (60%) LDs had a positive attitude regarding nutritional care of hyperphosphatemia-suffering HD patients. Lack of training and marginalization of them in the renal multidisciplinary team were pinpointed as the main reasons preventing their appropriate renal practice [43, 44].

This study has some limitations as well as strengths. Due to time and resource limitations, this study iwas conducted in only one governorate of Lebanon (Mount Lebanon), where the majority were young inexperienced females, and this may have underestimated the knowledge, attitudes, and dietetic practices of LDs. Therefore, the diverse results obtained cannot be generalized to the entire Lebanese population. It is exhorted that analogous studies take place in other Governorates with an expanded framework where wider HD complications are nutritionally tackled. The study was subject to some limitations including the questionnaire being self-reported, which may lead to response and social desirability bias, in addition to reporting bias. Recall bias is minimal as the questions pertaining to the knowledge, attitude and practice of dietitians regarding hyperphosphatemia of patients undergoing HD are of current status in nature, therefore no recall is needed by the dietitians to answer these questions. Since it was an online survey, there was an inability to control the possible referral of dietitians to outside sources to answer knowledge questions. The snowball sampling method was used to select from hospitals and private practices 408 dietitians having characteristics meeting the objectives of the present study requirements. This method allowed to recruit participants through referrals of original study participants which helped to ease the difficulty of data collection during post-COVID lockdowns. Probabilistic sampling wasn’t found feasible for data collection. Regarding strengths, this is the first study in Lebanon to explore the knowledge, attitude and practice of dietitians regarding hyperphosphatemia management of patients undergoing HD and their correlates. On the other hand, the sample size adopted gives us the statistical power to derive credible and representative conclusions for the Mount Lebanon population. Although the instrument isn’t yet validated with the Mount Lebanon population, it was derived using prior literature [19, 23–26], based on other validated questionnaires. In particular, the section on knowledge of dietitians was based on a mixture of two questionnaires [19, 25, 26] which was modified to meet the study objectives while the section on sociodemographics was adapted from another questionnaire [27]. A multicentre observational study on phosphorus nutritional knowledge among dialysis health care providers and patients in Greece [13] also adapted the same questionnaires as ours.

## Conclusion

Despite limitations, the present study is a progenitor in proposing a key resolution for the improvement of renal nutrition care and the dietetic career in Lebanon among LDs working in both hospital and private settings. Some of the recommendations of this study reflect what was suggested in another study on renal nutrition knowledge of LDs [2] and the other examining the effect of nutrition education [3]. However, the first was limited to hospital settings and restricted by a small sample while the second focused on the management of osteodystrophy among HD patients in hospital settings. As a leap forward in this mission to guarantee fineness in quality therapy for HD patients and their relatives, we suggest the next work plan: train hospital dietitians and hospital dietetic interns on the KDOQI updated version, endorse renal turnover in internships, endorse the official guidelines that should be followed during the training period, and strengthen their execution and application, constant analysis, and evaluate the compliance with KDOQI guidelines in renal therapy [45–47], consider guidelines’ cooperation with nephrologists and renal nurses, unite resources that should be used, develop more renal nutrition education in universities, internships, clinical and hospital fields of work, consider more time for renal-HD training time in hospitals [48], consider more time for patients undergoing HD care by recruiting a specialized fully devoted dietitian for this delicate section [49]. Last, but not the least, consider more free online training and seminars [50] under the supervision of the syndicate of dietitians in Lebanon and the MOPH in the current hard times in Lebanon until the procurement of the necessary modifications in the nutrition curriculum. Therefore, the MOPH is advised to encourage the integration of nutrition programs, and aid in the authorization of renal dietitians in the medical team seeking the optimal professional care team for the most cost-efficient outcome of end-stage renal disease patients in Lebanon.

## Supplementary Information

Supplementary Material 1.

## Data Availability

The data is not publicly available. it may be obtained from Elissa I upon special request.
