# Supplementary material for: Knowledge, attitudes, and practices of Lebanese licensed dietitians regarding hyperphosphatemia management in patients undergoing hemodialysis in a Lebanese Governorate
Source: BMC Nephrol. 2025 Feb 14;26:81. doi: 10.1186/s12882-024-03936-w (PMC11829401; doi:10.1186/s12882-024-03936-w)
Supplement: Supplementary file 1 — Supplementary Material 1. [file 12882_2024_3936_MOESM1_ESM.docx]

**Appendix 1: Questionnaire for knowledge, attitudes, and dietetic practices of Lebanese licensed dietitians regarding hyperphosphatemia-suffering haemodialysis patients**

I am directed to assess nutritional phosphorus knowledge (cooking methods, food preparation), attitudes of all licensed dietitians, and the conformity of clinical practices with the national kidney foundation NKF-KDOQI guideline for nutrition in chronic kidney disease: 2020 update. And to identify the factors that prevent licensed dietitians from dealing with renal patients, because colloquium holders are entitled to do so.

Part I - Sociodemographic Information

This section contains questions about you that will allow us to describe our sample as well

as compare your experiences with others.

1. Gender

 Male

 Female

2. Age

 21 - 34 years

 35 - 44 years

 45 - 54 years

 Older than 55 years

3. Level of education

 Bachelor Degree BS

 Master degree MS

 PhD degree

4. Professional status

 Licensed dietitian (LD)

 Registered dietitian (RD)

5. Work field

 Clinical dietitian

 Hospital-based or H emodialysis-based dietitian

 Food service

 Public health or community nutrition (NGO’s, education)

 Freelance or working from home (Diet plans for friends and family…)

 Others (Research, administrative dietitian, sales…)

6. Years of experience

 0-2

 3-5

 6-10

 More than 10

7. Average Monthly Income

 Less than 675,000 LBP

 Between 675,000 LBP and 1,500,000 LBP

 Between 1,500,000 LBP and 3,000,000 LBP

 Between 3,000,000 LBP and 5,000,000 LBP
 More than 5,000,000 LBP

Part II – KAP Questionnaire on knowledge, attitudes and practices of Lebanese registered dietitians regarding hyperphosphatemia-suffering hemodialysis patients.

I- Knowledge

This part is divided in three sections: knowledge, attitudes and practices. The first section contains questions about your knowledge on kidney disease, with a special focus on hyperphosphatemia, diet, and phosphate binders. The following questions concern all licensed dietitians from all areas of work because a colloquium holder is entitled to treat a renal patient.

1. Is chronic renal failure caused by a progressive decline in all kidney functions,

ending with terminal kidney damage?
Yes

 No

 Don’t Know/I’m not sure

2. Is there any cure for end-stage renal disease (ESRD) or kidney failure?

 Yes

 No

 Don’t Know/I’m not sure

3. Is hemodialysis the only treatment for end-stage renal disease?

 Yes

 No

 Don’t Know/I’m not sure

4. Which of the following problems may result from high phosphorus load??

 Liver disease

 High blood pressure and cardiac disease

 Bone and joint disease

5. What happens when phosphorus levels are high in blood? Calcium will be pulled out of bones

 Osteodystrophy

 Calcium levels in blood will drop

 All of the above

6. What would high blood phosphorus level lead to?

 Heart arrhythmia

 Muscle cramp

 Dizziness

 Itchy skin

7. Why does a hyperphosphatemia-suffering hemodialysis patient need to avoid all food items that are rich in phosphorus?

 Because the liver is incapable of removing the excess phosphorus from the blood

 Because the kidney is incapable of removing the excess phosphorus from the

blood

 Food items rich in phosphorus should consumed abundantly

8. Which of the following foods contain high amounts of phosphorus?

 Sesame seeds, sesame paste, halawa

 Nuts & seeds

 Organ meats, Liver, sausage

 Lentils, chick peas, white kidney beans

 All of the above

9. What are phosphate binders?

 Medicine that decreases the absorption of potassium from food (in stomach) to the blood

 Medicine that decreases the absorption of calcium from food (in stomach) to the blood

 Medicine that decreases the absorption of phosphorus from food (in stomach) to the blood

 Medicine that decreases the absorption of magnesium from food (in stomach) to the Blood

10. What is the right time to give hemodialysis patients phosphate binders?

 Between meals

 8:00 – 13:00 – 18:00

 With every time he/she eats a meal

 Before every meal

11. Which of the following foods contain low amounts of phosphorus?

 Bread sticks without sesame seeds

 Honey, Apple jam, quince jam

 Coffee mate

 All of the above

12. Which of the following drinks contain low amounts of phosphorus?

 Pepsi/Cola

 Lemonade, Seven Up/Sprite, Crush / Miranda

 Red Bull, Coffee – Nescafe

 Tea (not dark color), Mint tea, Anise tea

 B and D

13. Which of the following sweets are poor in phosphorus?

 Rice milk, pudding (made from milk) Custard

 Jell-O, Sorbet, biscuits

 “Kunafa” with cheese (Arabic sweet)

 Chocolate – Chocolate cake

 Milk based ice-cream

14. Which of the following food items are rich in phosphorus?

 String beans or green beans

 Milk and yogurt

 White bread and rice

 Tomatoes

15. Which of the following are optimal ways to control an adequate blood phosphorus level?

 Take phosphate binders regularly

 Commitment to a low phosphorus diet

 Starving oneself

 A & B

16. Which of the following food items are rich in phosphorus?

 Full fat milk and yogurt

 Low fat milk and yogurt

 Fat free milk and yogurt

 All have the same quantity

17. Is the phosphorus pyramid considered a tool for dietary phosphate management in hemodialysis patients?

 Yes

 No

 Don’t Know/I’m not sure

 In the phosphorus food pyramid, are the foods categorized into five levels on the

basis of: dietary phosphorus content, bio-availability and processing?

 Yes

 No

 Don’t Know/I’m not sure

18. What is/are the main strategy/strategies that may reduce dietary phosphorus intake?

 Boiling vegetables

 Boiling animal-derived products

 None of the above

 All of the above

19. What are the beverages and food’s phosphate additives?

 E340-E349

 E450-458

 All of the above

20. Which of the following food are classified as very low bio-available phosphorus?

 Egg white

 Fruits and vegetables

Protein-free products

 All of the above

21. What is the optimal level for blood phosphorus?

 Less than 6 mg/dl

 Higher than 8mg/dl

 Less than 1mg/dl

22. Who is responsible in controlling phosphate blood levels in the first place?

 The patient

 The doctor

 The dietitian

II – Attitudes

This section contains questions about your attitude if you have been consulted by a hemodialysis patient. The following questions concern all licensed dietitians from all work fields because a colloquium holder is entitled to treat a renal patient.

|  | Yes | No | Don’t Know/  I’m not sure |
| --- | --- | --- | --- |
| 1. If you have been consulted by a hemodialysis patient, would you agree to take nutritional care of this case? |  |  |  |
| 2. Have you ever been consulted by a hemodialysis patient but you have referred her/him to another dietitian or a renal specialized one? |  |  |  |
| 3. Have you ever referred this patient to another dietitian but this dietitian apologized and asked you to refer it to another renal specialized dietitian? |  |  |  |
| 4. Do you think that shortage lack of knowledge, lack of motivation, fear from “renal case”, incapacity of handling hemodialysis patient due to lack of trainings and information were one of the main reasons for referring the patient to another dietitian? |  |  |  |
| 5. Do you think that shortage in data available and don’t know which resource to adapt (KDOQI, Krause), confusion concerning referral resources were one of the main reasons for referring the patient to another dietitian? |  |  |  |
| 6. Do you think that shortage in data adapted to Lebanese Community concerning high and low phosphorus food for example, was one of the main reasons for referring the patient to another dietitian? |  |  |  |
| 7. Do you think that licensed dietitians are marginalized in renal therapy? (If no or don’t know → please skip question 8) |  |  |  |

|  | Yes | No | Don’t Know/  I’m not sure |
| --- | --- | --- | --- |
| 8. Since dietitians are the most marginalized in renal therapy, lack of cooperation with nephrologists, contradiction between dietitians and nephrologists opinions were one of the main reasons? |  |  |  |

III – Practices

This section contains questions about your clinical practice if you have been consulted by a hemodialysis patient and pre-assuming what would you do in this case. We simply want to learn how you, a front-line nutrition professional, currently practice despite not always having established guidelines for the issues you encounter. Please answer what you would do, i.e., or what you do about 80% of the time.

1. In your practice, what is the best way to initially assess the nutritional status for hemodialysis patients?

 7-Point Subjective global assessment (SGA)

 Malnutrition inflammation score (MIS)

 All of the above

2. In your practice, which method of body composition assessment is the most valid and the most reliable tool for hemodialysis patients?

 Multi-frequency bioelectrical impedance

 Dual energy X-ray absorptiometry

 None of the above

3. In your practice, which alternative method(s) do you use to assess dietary intake for hemodialysis patients?

 3-day food record

 24-hour food recalls

 Food frequency questionnaires

 nPCR

 B, C & D

4. In your practice, next to dietary intake, what are the factors that should be taken into consideration when assessing dietary intake of hemodialysis patients?

 Depression

 Behavior

 Access to food and knowledge

 Cognitive function

 Depression and behavior

 All of the above

5. What are the daily protein requirements for hemodialysis patient suffering from diabetes?

 1.0-1.2g/kg/day body weight

 0.8-1 g/kg/day body weight

 0.6-0.8 g/kg/day body weight

6. What are the daily energy requirements for hemodialysis patients who are

metabolically stable?

 20-30 kcal/kg body weight per day

 25-35 kcal/kg body weight per day

 40 kcal/kg body weight per day

7. In your practice, is it reasonable in collaboration with the physician, to consider

multivitamin supplementation for individuals with adequate vitamin intake?

 Yes

 No

 Don’t know/I’m not sure

IV- Interest in renal specialization in Lebanon

This section contains questions about your interest in renal specialization. Also, it will help us identifying reasons that prevent dietitians from dealing with renal patients.

1. Are you interested in renal specialization?

 Yes

 No → Skip question 2, 3, 4 and 5

2. You wanted to continue your degree in renal specialization in Lebanon, but couldn't due to lack of specialization?

 Yes

 No

3. You took the initiative to register in an online renal course, trainings, and seminars?

 Yes

 No → Please skip question 4 and 5

4. How many dialysis patients have you been or you are now responsible for (Panned a diet and did a follow-up with)?

 0 → Skip question 5

 1-10

 11-25

 26-50

 51-100

 101-150

 151 or more

5. What was the source of patient education material that you used?

 KDOQI reference

 Krause reference

 Dietitian prepared it with colleagues

 Dietitian prepared it alone

 No tool

6. Do you think a bigger focus should be done on renal disease management in the

nutrition curriculum: Renal specialization should be incorporated in Lebanese

universities curriculum?

 Yes

 No

7. In your opinion, and given that you’re entitled to do so, what are the reasons that

are preventing you to deal with hemodialysis patients?

 Lack of knowledge

 Lack of trainings

 Lack of time (work-overload if you have been a hospital-based dietitian)

 Marginalization of renal therapy in universities

 Marginalization of dietitians in the renal multidisplinary team (Nurses and

nephrologists)

 Fear of renal “cases” since its one of the most complicated diseases

 Lack of economic resources leading to incapacity of pursuing renal specialty abroad

 All of the above

 Other reasons (Specify)

 I don’t know

**Thank you for completing this questionnaire.**

**Your assistance is very much appreciated.**
